# The Occurrence of Illicit Smart Drugs or Nootropics in Europe and Australia and Their Associated Dangers: Results from a Market Surveillance Study by 12 Official Medicines Control Laboratories

**DOI:** 10.3390/jox15030088

**Published:** 2025-06-06

**Authors:** Celine Vanhee, Eric Deconinck, Mark George, Andrew Hansen, Andreas Hackl, Uwe Wollein, Oliver El-Atma, Nico Beerbaum, Federica Aureli, Anna Borioni, Magdalena Poplawska, Agata Blazewicz, Karin Roschel, Claude Marson, Magnolia Mendoza Barrios, Birgit Hakkarainen, Andreas Blomgren, Ingrid Bakker-‘t Hart, Marta Miquel

**Affiliations:** 1 Scientific Direction Chemical and Physical Health Risks, Service of Medicines and Health Products, Sciensano, J. Wytsmanstraat 14, 1050 Brussels, Belgium; 2 Therapeutic Goods Administration Laboratories, Australian Government Department of Health and Aged Care, P.O. Box 100, Woden, ACT 2606, Australia; 3 AGES—Österreichische Agentur für Gesundheit und Ernährungssicherheit GmbH, Spargelfeldstraße 191, A-1220 Wien, Austria; 4 Department of Pharmacy (OMCL), Bavarian Health and Food Safety Authority LGL—Bayern, Veterinärstraße 2, 85764 Oberschleißheim, Germany; 5 State Institute of Chemical and Veterinary Analysis Karlsruhe, Weißenburger Str. 3, 76187 Karlsruhe, Germany; 6 Department Lebensmittel und Arzneimittel, Landeslabor Berlin-Brandenburg-Institut für Lebensmittel, Arzneimittel, Tierseuchen und Umwelt, Rudower Chaussee 39, 12489 Berlin, Germany; 7 Nazionale Controllo e Valutazione dei Farmaci Reparto Farmaci Chimici, Direttore, Istituto Superiore di Sanità, Viale Regina Elena 299, 00161 Roma, Italy; 8 National Medicines Institute, 30/34 Chelmska Str., 00-725 Warsaw, Poland; 9 Laboratoire National de Santé, Rue Louis Rech 1, L-3555 Luxembourg, Luxembourg; 10 Chemical and Pharmaceutical Division, Medicines for Human Use Department, Agencia Española de Medicamentos y Productos Sanitarios (AEMPS), Calle Campezo 1, Edificio 8, E-28022 Madrid, Spain; 11 Swedish Medical Products Agency, Box 26, Dag Hammarskjölds väg 42, SE-751 03 Uppsala, Sweden; 12 National Institute for Public Health and the Environment, P.O. Box 1, 3721 MA Bilthoven, The Netherlands; 13 The European Directorate for the Quality of Medicines & HealthCare, Council of Europe, 7 allée Kastner, CS 30026, F-67081 Strasbourg, France

**Keywords:** illegal medicinal products, medicines in disguise, nootropic drugs, unauthorised novel food, research chemical

## Abstract

In recent years, an increasing number of case reports have mentioned the presence of illicit nootropics, smart drugs or mind doping products on the market. To better understand the extent of the problem, a market surveillance study was organised by the General European Official Medicines Control Laboratory Network and associated member Australia to detect substandard, falsified or illegal medicines or dietary supplements containing unauthorised nootropic molecules of natural or synthetic origin. From January 2020 to September 2024, 159 different samples were documented, which yielded a comprehensive dataset of 166 molecular identification entries. Within this dataset, 34 distinct molecules were identified. Most samples were sold or presented as dietary supplements (49%) or medicines (32%). The vast majority (69%) were collected from the illegal market. Prescription drugs and non-authorised drugs only available on prescription in Russia were found in pharmacological quantities; some of the latter (noopept, phenylpiracetam and phenibut) were intercepted as large bulk quantities of raw material. Unauthorised novel foods, prescription or higher amounts of melatonin, and clinically uncharacterised research molecules were also reported. This study highlights the need for more active monitoring and screening of such products, as consumption of some of the reported samples could have detrimental health effects. Furthermore, as a large number of the samples were presented as dietary supplements, consumers may not be aware of the possible dangers and side-effects of these products.

## 1. Introduction

The demand for health products containing nootropics, smart drugs, brain boosters, or cognitive enhancers has skyrocketed in recent decades, reflecting the growing interest in enhancing cognitive function, focus, and overall mental well-being to cope with a fast-paced society [1,2,3,4,5]. This market includes not only health products claiming to boost or aid mental performance but also products aiming to promote a better quality of sleep, mood regulation, stress relief, and overall brain health. In recent years, several reports have emerged, describing the adulteration of purported nootropic health products, often presented as dietary supplements. These items were either adulterated with nootropic pharmaceuticals or contained unauthorized food with purported nootropic effects, unapproved or banned pharmaceuticals, and even research chemicals for which limited or no pharmacological and/or toxicological data are available [5,6,7,8,9,10,11,12]. Substandard and falsified (SF) medicines, containing prescription-only medicines such as methylphenidate, modafinil and high doses of melatonin, often purchased from the internet, were also reported to be used for their nootropic effects [5,13,14,15,16]. These illicit nootropics are used by a variety of consumers, including university students, white-collar workers, night shift workers, e-sports players, and the elderly [5]. In the European Union (EU), the Official Medicines Control Laboratories (OMCLs) support the regulatory authorities in controlling the quality of medicinal products available on the market. This testing is conducted independently from manufacturers, thus without any conflict of interest. Some OMCLs also perform the testing of illegal medicines and para-pharmaceutical products such as dietary supplements. These OMCLs are not restricted to the EU and are also located in several non-European countries, including Australia. In the EU and Australia, dietary supplements are regulated differently compared to medicines, as the main responsibility resides not with the national competent authority but with the food business operator to make sure that the food is safe [17]. However, the European Food Safety Authority (EFSA) issues advice on existing and emerging food risks, which can then impact European laws, rules, and policymaking. This was well illustrated by the ban on *Ephedra* and the bark of *Pausinystalia yohimbe* and preparations thereof in food, including dietary supplements [18]. In addition, if a food ingredient has not been consumed to a significant degree by humans in the EU before 15 May 1997, the food business operator should first apply to the European Commission, which then requests a scientific assessment from EFSA [19]. If the EFSA panel of experts has safety concerns, the novel food ingredient will not be authorised. A similar procedure is also in place in Australia [20]. In certain countries, OMCLs or other laboratories also check for the presence of unauthorised novel foods in dietary supplements, as is sometimes the case for nootropics.

The aim of this study was to quantify and characterise the prevalence of illicit nootropics in the European Union and Australia through a coordinated market surveillance effort. Specifically, we sought to identify substandard and falsified medicines, dietary supplements containing unauthorised molecules of either natural or synthetic origin, and products containing research chemicals with unknown safety profiles. This research represents the first multi-country investigation of the illicit nootropics market conducted by the General European OMCL Network (GEON) and its associated member Australia, building upon previous successful surveillance studies targeting other categories of problematic health products [21,22,23,24,25,26,27,28].

## 2. Materials and Methods

### 2.1. Scope

**Figure 1:**
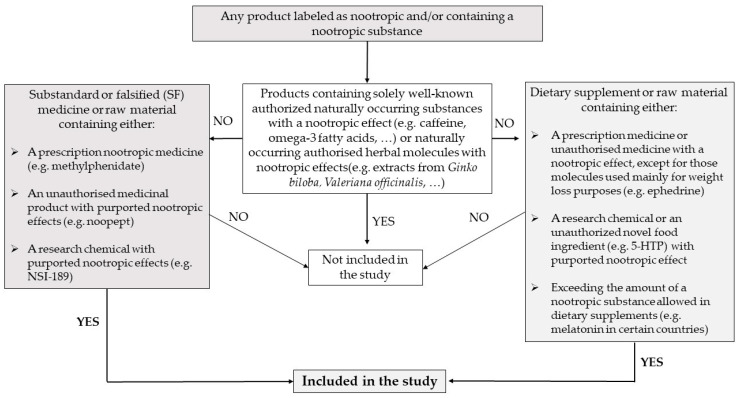
Decision tree illustrating whether or not a sample should be included into the study.

The scope of the study was defined as any product (medicine, herbal medicine, dietary supplement, raw material, etc.) labelled as nootropic and/or containing an illicit nootropic substance. The nootropic substances comprise all substandard and falsified (SF) medicines containing an active pharmaceutical ingredient (API), unauthorised medicinal products, nootropic research chemicals, and semi-synthetic molecules with nootropic effects (e.g., vinpocetine). SF medicines are defined by the World Health Organization (WHO) as either authorised medical products that fail to meet their quality standards or specifications (substandard) or medical products that deliberately/fraudulently misrepresent their identity, composition, or source (falsified medicines). In addition, all dietary supplements or raw materials containing a nootropic prescription medicine, a banned substance with nootropic properties, a nootropic research chemical, or an unauthorised novel food nootropic ingredient, or exceeding the amount of certain nootropic substances in dietary supplements (e.g., melatonin) were included in the study (see Figure 1). If a medicinal product or dietary supplement was positive for melatonin, the quantity present determined whether the sample was included or not. In Australia and some EU countries, it is a prescription drug and thus not allowed in dietary supplements. The limit for over-the-counter (OTC) melatonin products and the amount that can be present in dietary supplements is country-dependent. In Luxembourg and Belgium, melatonin can be present in dietary supplements up to a daily intake of 0.3 mg, up to 1 mg in Italy, Spain, and Poland, and up to 2 mg in France. If no quantification for melatonin was performed, the sample was not included in the study. Products containing well-known naturally occurring authorised substances with a nootropic effect (e.g., astaxanthin, berberine, caffeine, carnosine, choline, creatine, fisetin, glutathione, glycine, omega-3 fatty acids, piperine, resveratrol, taurine, theanine, theobromine and quercetin) or naturally occurring authorised herbal molecules with nootropic effects originating from *Ginko biloba*, *Melissa officinalis*, *Magnolia officinalis*, *Panax ginseng*, *Passiflora incarnata*, *Piper methysticum*, *Polygala Tenuifolia*, *Rosmarinus officinalis*, Sage, *Theobroma cacao*, and *Valeriana officinalis*, for example, were considered to be outside the scope of the present study.

Products containing cocaine, amphetamine and analogues sold or purchased as party drugs were also considered outside the scope of the study, although products containing these molecules sold for their nootropic effects were included. As well, samples that contained herbal-based stimulants (e.g., (pseudo)ephedrine, synephrine) but mainly sold as weight loss enhancers were also not incorporated into the study. Moreover, samples that claimed to contain nootropics but did not were also excluded from this study.

### 2.2. Data Collection and Time Frame

The different participating OMCLs analysed the suspect products according to their standard operating procedures. Most of them identified the majority of the APIs in these products using a chromatographic approach hyphenated with (high resolution) mass spectrometry. The most frequently used strategies are summarized in the position paper “An aide-memoire for the testing of suspected illegal traded and falsified medicines” [28]. The results for the samples falling within the scope of the study were reported in a harmonized way using a Microsoft Excel template as previously described [16].

The study was a combined retro- and prospective study covering a time frame from January 2020 to the end of September 2024.

## 3. Results

The participating OMCLs reported 159 different samples that fit into the predefined scope and can be found in Table S1. A list of the different molecules detected, their prevalence and their current legal status can be found in Appendix A.

### 3.1. Origin of the Samples

**Figure 2:**
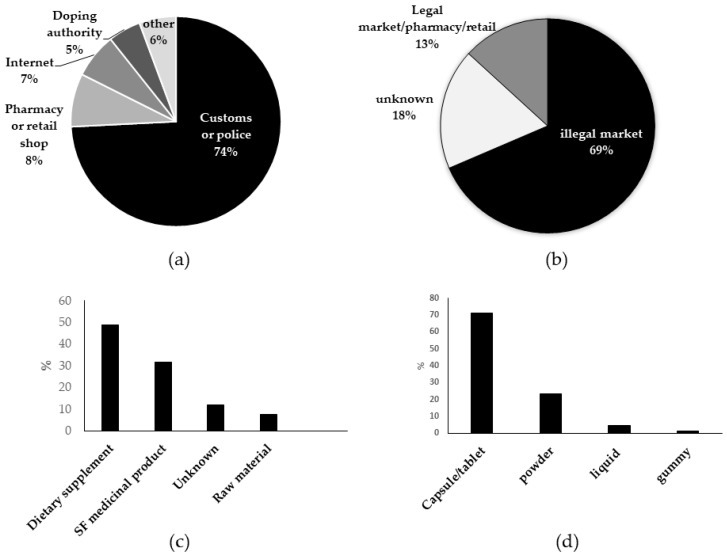
Origin of the samples (**a**), percentage of samples originating from the legal or illegal market (**b**), the form in which they were presented to the consumer/patient (**c**), and their dosage forms (**d**).

More than 74% of the samples came from customs or law enforcement agencies, 5% from doping agencies, and only 8% from routine inspections. Of the remaining samples, almost 7% were reported to originate from the internet (see Figure 2a). It should be noted that the samples reported here are the ones analysed by the participating OMCLs. In the context of law enforcement and customs control, in the majority of countries, samples are also analysed by other laboratories linked to justice or customs. This means that more samples are likely to be intercepted and analysed in different countries than those in the sample set featured in this study.

Upon further analysis of the samples, it was found that the majority of the samples (69%) originated from the illegal supply chain, while 13% came from the legal supply chain (pharmacies and retail shops), as can be seen in Figure 2b. The latter products mainly concerned the presence of unauthorised novel food ingredients (e.g., 5-hydroxytryptophan (5-HTP)) or exceeding of the maximum tolerated level of melatonin in dietary supplements. These findings are not necessarily surprising, as non-conformities in dietary supplements available in the EU are often reported through the Rapid Alert System for Food and Feed (RASFF) [29,30] and in the scientific literature [31,32,33,34,35,36]. For the remaining 18% of the sample set, the reporting OMCL could not pinpoint whether the sample originated from a legal or illegal supply chain.

### 3.2. Product Type and Dosage Form of Tested Samples

The majority of the samples were either sold or presented as a dietary supplement (DS 49%) or as a medicine (32%), as illustrated in Figure 2c. The remaining samples were sold or presented as large bags of raw material (8%), or the OMCL in question was not able to determine how the material was presented to the patient or consumer. The different reported dosage forms of the samples are given in Figure 2d. It can be noted that the majority of the samples were either capsules or tablets (≈71%), powders (24%), liquids for either oral or nasal use (4%), or gummies (1%). This finding is not necessarily surprising, as these nootropic molecules are often taken orally.

### 3.3. Detected Molecules and Their Quantities

**Figure 3:**
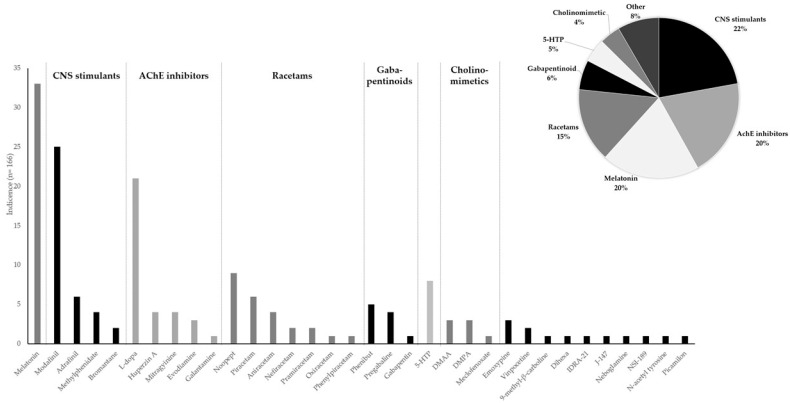
Abundance of the different nootropic molecules found and their classification based on their (assumed) functional activity and the percentage found. Abbreviations: DMAA = dimethylamylamine; DMPA = 1,4-dimethylpentylamine; 5-HTP = 5-hydroxytryptophan; L-Dopa = levodopa.

The participating OMCLs reported 34 unique molecules, as can be seen in Table A1 and Figure 3. Table A1 also describes how the samples were presented and their legal status, which may differ among the different participating countries.

The top three reported molecules, melatonin, modafinil, and levodopa (L-Dopa), account for more than half of the total entries. These are well-known nootropics that are used either as a sleep medicine (depending on the amount of melatonin), a central nervous system stimulant (CNS) used to treat certain cases of narcolepsy, and in medication for Alzheimer’s disease (in combination with other molecules). In addition to proper identification, the amount present was of paramount importance for melatonin when considering whether to include samples in this study or not. The minimum dosage that was detected corresponded to 0.5 mg per capsule or tablet (see Table 1).

**Figure 4:**
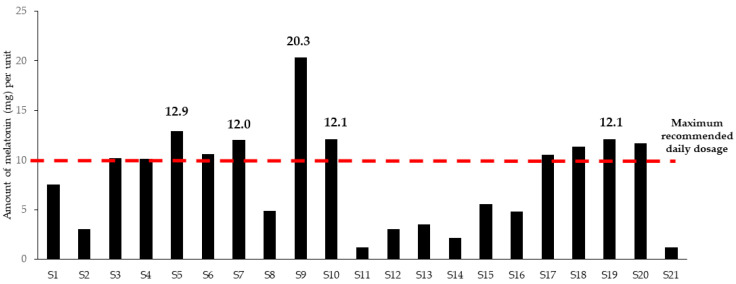
Amount of melatonin, expressed in mg per tablet or capsule (=unit), plotted for the different samples exceeding the national threshold allowed in dietary supplements. Several samples contain more than 10 mg of melatonin, corresponding to the maximum therapeutic dosage often used for short-term medicinal use by adults.

For melatonin, it was found that of the 35 samples, 21 exceeded the lowest therapeutic dosage (see Figure 4), while five samples exceeded the recommended maximum dosage of 10 mg a day by at least 20%. The maximum amount of melatonin in one dietary supplement corresponded to 20.3 mg per unit (capsule or tablet), which is double the recommended maximum daily dosage (see Table 1). The amount of modafinil found varied from 88 to 197.3 mg per unit, which represents a therapeutic dosage, as often, quantities of 100–400 mg per day are given (see Table 1).

The third most common molecule, L-Dopa, often encountered in dietary supplements containing the unauthorised novel food *Mucuna pruriens*, was present in quantities ranging from 1.2 to 40 mg per capsule or tablet. These amounts are lower than the pharmacological quantities often used in medicinal products (300 to 1200 mg per day, divided into 3–12 doses). These products also contain concomitant drugs to inhibit the peripheral metabolization of L-Dopa into dopamine and concurrent undesired side-effects such as nausea due to peripheral dopamine production [9]. Also, other acetylcholinesterase (AChE) inhibitors were also found, such as the prescription drugs galantamine [11] and huperzine A [37], the potential AChE inhibitor evodiamine [38], currently being studied for its anti-cancer properties, and the psychoactive compound mitragynine. The amount of galantamine found was about 4 mg per capsule or tablet, which, if taken twice a day, would correspond to a pharmacological dosage [11]. Molecules belonging to the AChE inhibitors accounted for 20% of the incidences, while CNS stimulants such as previously mentioned modafinil, modafinil prodrug adrafinil, methylphenidate, and bromantane accounted for 22% (see Figure 3). Based on the reported quantification data, adrafinil was present in pharmacological quantities (see Table 1).

In addition to AChE inhibitors and CNS stimulants, members of the racetam family were detected several times, with a total contribution of 15% of the reported molecules. Noopept was the most commonly detected and is currently used as a prescription medicine in Russia to treat traumatic brain injury, mood disorders and cerebral vascular disease [7]; however, its use has not been authorised in the EU or Australia. Often, noopept was presented or sold as raw material in bulk with a purity of up to 100% (see Table 2). One OMCL reported the amount of noopept corresponded to 20 mg/mL, which is twice the typical pharmacological oral dose used in Russia [7]. Piracetam and aniracetam were also detected. In the EU, piracetam is a prescription drug, while in Australia, piracetam is not authorised but aniracetam is. The recommended pharmacological maximum dosage of aniracetam corresponds to 750 mg a day. This value is very close to the maximum amount present in one sample (see Table 1). Several other piracetam homologues were also found, but with lower incidences. It should be noted that in the case of the homologue phenylpiracetam, bulk quantities of raw material were intercepted with purities close to 100% (see Table 2). Phenylpiracetam is available as a prescription medication in Russia with recommended pharmacological dosages of 100–200 mg/day [7].

Other drug classes such as the gabapentinoids, represented here by the prescription drugs gabapentin, phenibut and pregabalin, and the cholinomimetics, including dimethylamylamine (DMAA), 1,4-dimethylpentylamine (DMPA), and meclofenoxate or centrophenoxine, account for 6% and 4% of the incidences, respectively (see Figure 3). Gabapentin is a prescription drug in the EU and Australia; the pharmacological dosage depends on the symptoms to be treated, and daily dosages from 600 mg to 3.6 g are used. Based on this information, pharmacological amounts are present in the sample set. Phenibut is also a Russian prescription drug, not authorised in the EU or Australia, for which the typical pharmacological dosages range between 250 and 500 mg a day [8]. The minimum amount of phenibut found was 81 mg, and the maximum was 250 mg per capsule or tablet (see Table 2). As for the two other Russian prescription drugs noopept and phenylpiracetam, bulk amounts of raw material were intercepted. Meclofenoxate, a prescription medicine in some EU member states but not approved in others [10], was also found in pharmacologically relevant quantities (Table 2). The same is also valid for vinpocetine, which is also a prescription medicine in certain member states. Moreover, several research molecules, many for which only in vitro and animal data are available, were reported by several OMCLs, and they accounted for 8% of the samples (Table A1). Obviously, it is impossible to determine whether the amount found in these samples reflects a pharmacological dosage (Table 2). Lastly, 5% of the samples were positive for the unauthorised novel food commodity, 5-HTP. 5-HTP may boost serotonin levels and have a positive effect on mood, depression, anxiety, sleep, appetite, and pain [41,42], although high-quality clinical studies are lacking. There have been reports associating eosinophilia-myalgia syndrome with 5-HTP supplements that may have been contaminated [49,50]. The amount found ranged from 3 to 100 mg per unit, and the dosage described in old clinical studies ranged from 100 to 900 mg/day (Table 2).

## 4. Discussion

From the results of this study, it can be seen that illegal nootropics—in the form of SF medicines, unauthorised raw materials, dietary supplements adulterated with prescription drugs, unapproved drugs or banned substances, unauthorised novel food and even research chemicals that have not yet undergone clinical trial—are circulating in parts of Europe and Australia. A total of 159 different samples were documented, from which 34 distinct molecules were identified. The 13 participating OMCLs reported 159 different samples, 166 molecule identification entries and 34 unique molecules. This study has some limitations. Given that only a limited number of OMCLs participated in this study, and that the majority of samples originated from customs or law enforcement agencies—which also have their own laboratories—it is likely that this study only highlights a very small fraction of this booming market, and that many more illicit nootropics are in circulation, including in the form of illicit dietary supplements. Moreover, in some countries, dietary supplements are not analysed by the OMCL or are not often subjected to chemical analysis by regulatory entities when no adverse effect has been reported. Nevertheless, the results presented illustrate that illicit nootropics are circulating within Europe and Australia.

Previous studies from the United States of America have shown that some dietary supplements contained unapproved nootropic drugs or prescription drugs [7,8,9,10,11], as is also illustrated by the different warning letters sent out by the US Food and Drug Administration [51]. Our findings also show that a large number of the samples included in the study (48%) are presented or sold as dietary supplements. In this case, the consumer is likely not aware that they are taking a prescription medicine, an unapproved drug, an unauthorised novel food or even a research molecule for which little to no toxicological data are available. This certainly carries some risk, as the occurrence of any potential adverse effects will not immediately be linked to the supplement by the patient or by the healthcare professional. In addition, these illegally added substances may interfere with other medicines, with consequences for the development or treatment of existing diseases. A recent case report illustrated the dangers of using *Mucuna pruriens* supplements in combination with carbidopa/levodopa treatment for Parkinson’s disease, resulting in dopamine dysregulation syndrome and the patient’s hospitalisation [52].

In this study, nine different prescription drugs, each with their inherent contraindications and possible side-effects, were detected in pharmaceutically relevant quantities (see Table 1). In the case of melatonin, five of the 21 samples for which quantification data were available exceeded the recommended maximum daily amount of 10 mg. One sample contained 20 mg of melatonin. Although a life-threatening melatonin overdose is very rare in adults, high dosages could result in unwanted side-effects such as drowsiness, dizziness, fatigue, headache, confusion, nightmares, hypotension, tachycardia, and hypothermia [53]. Nevertheless, lethal melatonin overdoses have been reported in children [54]. Pharmacological doses of CNS stimulants such as modafinil, adrafinil, and the drug of choice for the treatment of attention deficit hyperactivity disorder (ADHD), methylphenidate, were also reported. Often, these drugs were present as SF medicines, and the unsupervised use of these prescription medicines is certainly not without risk, as they can result in severe side-effects, including rapid and irregular heartbeat, anxiety, hallucinations, thoughts of self-harm, and allergic reactions [55,56]. Pharmacologically relevant quantities of aniracetam, gabapentin, meclofenoxate, and vinpocetine were reported. Aniracetam is used to treat symptoms of hyperactivity, such as restlessness, and is generally well tolerated. The most common adverse events reported with aniracetam use are unrest, anxiety, uneasiness, and insomnia. Other unwanted side-effects include urinary urgency, headache, vertigo, mild stomach pain, nausea, diarrhoea, and rash [57]. The misuse or abuse of gabapentin, on the other hand, can lead to dependence with withdrawal symptoms comparable to those from benzodiazepines and alcohol: anxiety, insomnia, nausea, pain, excessive sweating, tremors, headaches, depression, feeling abnormal, dizziness and malaise [58]. For this reason, several healthcare authorities advocate for the gradual discontinuation of this product and pregabalin, a gabapentinoid also reported in this study (see Table A1 and Figure 3).

Alongside these prescription medications, unauthorised substances available only by prescription in Russia (such as noopept, phenylpiracetam, and phenibut) have been identified, often in substantial quantities of raw material. These findings raise significant concerns, indicating not just individual consumption but also potentially broader sales and trafficking activities, with products possibly compounded with harmful intent in the participating countries. The use of these Russian medications, including bromantane, carries risks as exemplified by the numerous reports focusing on the side-effects caused by phenibut, including coma, respiratory depression, and even fatalities [59]. Moreover, research chemicals with minimal or no clinical or toxicological information are being utilised in Europe and Australia and are being marketed online. Such products present a considerable danger to users and complicate healthcare professionals’ ability to manage adverse reactions. This risk extends to dietary supplements featuring unregulated or unapproved novel food ingredients like *Huperzia serrata* or *Evodia rutaecarpa*, which provide sources of huperzine A and evodiamine, respectively. A recent toxicological study conducted by the Dutch National Institute for Public Health and the Environment (RIVM) warns consumers against using herbal products containing *H. serrata* or its extracts due to reported harmful health effects, including unwanted gastrointestinal issues and paralysis [35]. Evodiamine, a bioactive alkaloid, exhibits various pharmacological effects such as anti-cancer, anti-bacterial, anti-obesity, anti-neurodegenerative, anti-depressant, and cardiac protective properties. Despite its potential health benefits, the clinical application of evodiamine is limited by its low water solubility, poor bioavailability, and toxicity [60,61]. Nonetheless, dietary supplements containing evodiamine are noted in this study.

This research reveals several significant implications for different stakeholders. Consumers should be made aware of the substantial risks associated with buying medicines or dietary supplements online, especially from dubious websites that lack proper authorisation from health authorities or conceal their physical identity and business details. For regulatory authorities and policymakers, our findings emphasize the urgent need for stronger monitoring systems for dietary supplements, including routine screening for illicit substances. Additionally, these results advocate for more publicly accessible, transparent, and unified regulations governing dietary supplements across EU member states, addressing the current lack of coherent guidelines [62].

## 5. Conclusions

Our study documented 159 samples containing 34 distinct molecules across European and Australian markets, revealing the circulation of illicit nootropics ranging from substandard and falsified medicines to adulterated dietary supplements and experimental research chemicals. With only 13 participating OMCLs, these findings likely represent only a small fraction of this expanding market.

Nearly half (48%) of the analysed samples were marketed as dietary supplements, which may mislead unaware consumers, as they are consuming prescription drugs or unknown compounds. We identified nine different prescription drugs in pharmacologically relevant quantities, including high-dose melatonin exceeding recommended limits, CNS stimulants (modafinil, methylphenidate), and medications like gabapentin and vinpocetine. The detection of unauthorised Russian prescription drugs (noopept, phenylpiracetam, phenibut) in bulk quantities suggests distribution networks that extend beyond personal use. Most concerning was the identification of experimental research chemicals for which no comprehensive clinical data are available or which are still undergoing preliminary trials in controlled studies, posing significant risks to users who may be unknowingly exposing themselves to substances with unpredictable effects, unknown toxicity profiles, and potentially dangerous long-term health consequences that have not yet been documented in the scientific literature. Moreover, unauthorised novel food ingredients with documented toxicity concerns or for which no information is available were found.

These findings underscore the dangers for consumers purchasing supplements online from unverified sources and highlight the importance of more robust monitoring systems and harmonised regulations for dietary supplements across EU member states.

## Figures and Tables

**Table 1:** Amount of melatonin and prescription drugs found in one unit (capsule or tablet), the purity of bulk quantities of raw material expressed in mass percentage and the therapeutic range often used for these molecules.

	Minimum (mg/Unit)	Maximum (mg/Unit)	Purity Raw Material % (*w*/*w*)	Therapeutic Range
Melatonin	0.5	20.3	55.1	1–10 mg/day
Modafinil	88	197.3	n.d.	100–400 mg/day
Adrafinil ^a^	97	293	n.d.	300–600 mg/day
Methylphenidate	25.5		n.d.	5 mg taken 2–3 times a day up to 60 mg/day
L-Dopa ^b^	1.2	40	n.d.	50 mg several times a day, up to 1.6 g/day
Galantamine	4.0		n.d.	4–12 mg twice a day
Piracetam	60	100	n.d.	1200–4800 mg/day
Aniracetam	0.2	735	n.d.	200–750 mg/day
Gabapentin	795		n.d.	600–3600 mg/day
Meclofenoxate	251.1		n.d.	50–400 mg/day
Vinpocetin	24		n.d.	5–20 mg taken 3 times daily

^a^ The prescription drug containing adrafinil has been discontinued since 2011 due to an unfavourable risk–benefit ratio. ^b^ L-Dopa is usually given in conjunction with an inhibitor of dopamine decarboxylase, to inhibit the side-effects of peripheral dopamine production, such as nausea. Abbreviation: n.d. not determined.

**Table 2:** Amount of the different unauthorised drugs or research molecules found in one unit (capsule or tablet), purity of bulk quantities of raw material expressed in mass percentage % (*w*/*w*), and relevant clinical data or therapeutic range used in countries where the medicines are or have previously been authorised.

**Molecule**	**Minimum (mg/Unit)**	**Maximum (mg/Unit)**	**Purity, % (*w*/*w*)**	**Clinical Data or Usage Information**
Noopept	20		100	Prescription medication, available in Russia, to treat traumatic brain injury, mood disorders, and cerebral vascular disease. The typical pharmacological dosage is 10 mg/day [7].
Oxiracetam	270.7		n.a.	A recent clinical trial with 800 mg oxiracetam twice daily for 36 weeks showed an increase in cognitive recovery after stroke [39].
Phenylpiracetam	n.a.		100	Prescription medication, available in Russia, promotes memory, increases concentration, is anti-depressant, anti-anxiety, and improves mood and physical performance. The typical pharmacological dosage consists of 100–200 mg/day [40].
5-HTP	3	100	n.a.	5-HTP may boost serotonin levels and have a positive effect on mood, depression, anxiety, sleep, appetite, and pain, although high-quality clinical studies are lacking. There have been reports associating eosinophilia myalgia syndrome with 5-HTP supplements that may have been contaminated. Dosages of 100–900 mg/day are described in clinical studies [41,42].
Phenibut	81	250	100	Prescription medication, available in Russia, to treat anxiety, insomnia, and other issues. The typical pharmacological dosage is between 250 and 500 mg/day [7]
Bromantane	33.2		n.a.	Prescription medication, available in Russia, to treat “neurasthenia”. The typical pharmacological dosage is 50–100 mg/day [43].
9- MBC	11.8		n.a.	No clinical data on humans were found. An in vitro study with astrocytes showed that this component could have neuroprotective and neuro-regenerative properties for dopaminergic neurons [44].
IDRA-21	12.1		n.a.	No clinical data on humans were found. Studies in different animals (rats and monkeys) suggest improved memory after oral intake [45,46].
J-147	3.68		n.a.	No clinical data on humans were found. Studies in different animals (rats and mice) suggest that oral intake improves memory and reduces anxiety [47].
NSI-189	19.1		n.a.	This compound is currently being studied in clinical trials (phase 2) for its possible anti-depressant activity and pro-cognitive effects [48].

Abbreviation: n.a.: not applicable.

## Data Availability

The original contributions presented in this study are included in this article. Further inquiries can be directed to the corresponding author.

## Supplementary Materials

The following supporting information can be downloaded at: https://www.mdpi.com/article/10.3390/jox15030088/s1, Table S1: Excel file with the complete data set described in this study.

## Appendix A

**Table A1:** List of molecules detected by the different laboratories, sorted by prevalence and current legal status.

Molecule Found	Detection Frequency	# Laboratories Where Detected	Mainly Presented as	Legal Status
Melatonin	33	7	DS	Melatonin regulation varies. In Australia and some EU countries, it is a prescription drug and thus not allowed in dietary supplements. The limit for dietary supplements containing melatonin is 0.3 mg per day in the Netherlands, Luxembourg, and Belgium, 1 mg in Italy, Spain, and Poland, and 2 mg in France. In Germany, the situation is unclear and depends on the court.
Modafinil	25	6	SF medicine and DS	Prescription medicine in the EU and Australia.
L-dopa	21	4	DS	Prescription medicine in the EU and Australia. The molecule is also present in the plant *Macuna pruriens*, an unauthorised novel food in the EU. In Australia, *Mucuna pruriens* is a permitted ingredient with L-dopa as a mandatory component with an allowed limit of 1 mg/kg or 1 mg/L.
Noopept	9	4	SF medicine and DS	Not authorised for human use by any health authority in the countries where the molecule was found.
5-HTP	8	4	DS	Unauthorised novel food in the EU. In Australia, 5-HTP is regarded as a derivative of the prescription medicine tryptophan. Any product for human therapeutic use with a label claim of greater than 100 mg 5-HTP per recommended daily dose would be regarded as a prescription medicine.
Adrafinil	6	2	SF medicine and DS	Discontinued medicinal product, formerly a prescription medicine (prodrug of modafinil).
Piracetam	6	3	SF medicine and DS	Prescription medicine in the EU and Australia.
Phenibut	5	4	DS	Not authorised for human use by any health authority in the EU or Australia, registered as a controlled substance.
Aniracetam	4	3	SF medicine	Prescription medicine in the EU and Australia.
Huperzine A	4	2	DS	Huperzine A is extracted from *Huperzia serrata* and is considered an unauthorised novel food in many EU member states, with some exceptions (e.g., Belgium, France and Romania). It is not approved for use in medicines in Australia.
Methylphenidate	4	1	SF medicine	Prescription medicine in the EU and Australia
Mitragynine	4	4	SF medicine and DS	Found in *Mitragyna speciosa.* The plant is considered an unauthorised novel food in the EU. In some EU member states and Australia, it is considered a controlled substance
Pregabaline	4	1	SF medicine	Prescription medicine in the EU and Australia
Emoxypine	3	2	SF medicine and DS	Research chemical for which little reliable clinical information is available, thus not authorised for human consumption in the EU or Australia.
DMAA	3	3	DS	Banned in dietary supplements in the EU and Australia
DMPA	3	2	DS	Not authorised in dietary supplements in the EU or Australia
Evodiamine	3	1	DS	Research chemical. The molecule can be extracted from the dried, nearly ripe fruit of *Evodia rutaecarpa,* an unauthorised novel food in the EU and not approved for use in Australia.
Bromantane	2	2	unknown	Research chemical in the EU and Australia, used as a prescription drug in Russia
Nefiracetam	2	1	SF medicine	Piracetam homologue, not authorised for human use by any health authority in the EU or Australia
Pramiracetam	2	1	SF medicine	Piracetam homologue, not authorised for human use by any health authority in the EU or Australia
Vinpocetine	2	2	SF medicine	Prescription medicine in certain EU member states (e.g., Poland and Germany), unauthorised in others and in Australia
9-MBC	1	1	unknown	Research chemical
Meclofenoxate	1	1	unknown	Prescription medicine in some EU member states (e.g., Austria and Germany) and in Australia, not approved in others
Dihexa	1	1	DS	Research chemical
Gabapentin	1	1	SF medicine	Prescription medicine in the EU and Australia
Galantamine	1	1	Unknown	Prescription medicine in the EU and Australia
IDRA-21	1	1	Unknown	Research chemical
J-147	1	1	Unknown	Research chemical
N-acetyl tyrosine	1	1	DS	Unauthorised novel food ingredient
Neboglamine	1	1	SF medicine	Research chemical
NSI-189	1	1	Unknown	Research chemical
Oxiracetam	1	1	Unknown	Piracetam homologue, not authorised for human use by any health authority in the EU or Australia
Phenylpiracetam	1	1	SF medicine	Piracetam homologue and not authorised for human use by any health authority in the EU or Australia
Picamilon	1	1	DS	Research chemical in the EU and Australia, used as a prescription drug in Russia

Abbreviations: DMAA = dimethylamylamine; DMPA = 1,4-dimethylpentylamine; DS = dietary supplement; 5-HTP = 5-hydroxytryptophan; L-Dopa = levodopa; 9-MBC = 9-methyl-β-carboline; SF = substandard and falsified. # = number.
